# Potential traditional Chinese medicines with anti-inflammation in the prevention of heart failure following myocardial infarction

**DOI:** 10.1186/s13020-023-00732-w

**Published:** 2023-03-17

**Authors:** Zhen Zhang, Fei Chen, Jingjing Wan, Xia Liu

**Affiliations:** Department of Clinical Pharmacy, School of Pharmacy, Second Military Medical University, No. 325 Guohe Road, Yangpu District, Shanghai, 200082 China

**Keywords:** Myocardial infarction, Inflammation, Traditional Chinese medicine, Heart failure

## Abstract

Inflammation plays an important role in the development of heart failure (HF) after myocardial infarction (MI). Suppression of post-infarction inflammatory cascade has become a new strategy to delay or block the progression of HF. At present, there are no approved anti-inflammatory drugs used to prevent HF following MI. Traditional Chinese medicine (TCM) has been used clinically for cardiovascular disease for a long time. Here, we summarized the recent progress about some TCM which could both improve cardiac function and inhibit inflammation in patients or experimental models with MI or HF, in order to provide evidence for their potential application in reducing the onset of HF following MI. Among them, single Chinese medicinal herbs (eg. Astragalus and Salvia miltiorrhiza) and Chinese herbal formulas (eg. Gualou Xiebai Decoction and Sini Tang) are discussed separately. The main targets for their anti-inflammation effect are mainly involved the TLR4/NF-κB signaling, as well as pro-inflammatory cytokines IL-1β, IL-6 or TNF-α. It is worthy of further evaluating their potential, experimentally or clinically, in the prevention or delay of HF following MI.

## Background

Myocardial infarction (MI) is the most common cause of heart failure (HF). Roughly 2.5 million patients suffer from MI each year, and the incidence of HF after MI is approximately 25–40% according to the epidemiological studies [1, 2]. HF could occur at the time of MI onset (approximately 12–20.4% at admission), during hospitalization (approximately 4–39%), or after discharge from the hospitalization (approximately 13% at 30 days, 20–30% at 1 year and 1.3–2.2% per year afterwards) [3]. HF complicating MI significantly worsen the prognosis of patients. A registry-based study from the FAST-MI (French registry of Acute ST elevation or non-ST-elevation myocardial infarction) reported that 37.5% of acute MI patients developed HF, and these patients had a significantly increased risk of death during hospitalization (12.2% vs. 3.0%) and at 1 year (26.6% vs. 5.2%) when compared to MI patients without HF [4].

Although the causes of HF vary, neurohormonal blockade has been a foundational pharmacological therapy. According to the guideline of 2022 AHA/ACC/HFSA, currently first-line drugs include angiotensin receptor neprilysin inhibitor (ARNIs)/angiotensin-converting enzyme inhibitors (ACEIs)/angiotensin receptor blockers (ARBs), mineralocorticoid receptor antagonists (MRAs), and. beta-blockers [5]. Early (within 24 h) initiation of ACEIs and early (within 7 days) use of aldosterone antagonists are recommended for the prevention and treatment of HF following MI, while early use (< 24 h) of beta-blockers is not suggested due to an increased risk of cardiogenic shock and death [6]. Although there is a significant improvement in the survival of HF during past 30 years, the 5-year mortality is still approximately 50% [7]. These raise the clinical urgency of finding new therapeutic strategies to block or delay the development of HF following MI [8].

## Inflammation plays an important role in the development of HF following MI

Adverse cardiac remodeling is a basic pathological process during the progression of HF after MI, which has been widely recognized and reviewed [9, 10]. Ischaemia-induced tissue damage initiates a series of events affecting the heart’s normal structure and function, finally leads to adverse remodeling and HF. Many factors are involved in the process, including changes in left ventricular geometry, ischaemia/reperfusion injury and reactive oxygen species, myocardial hypertrophy and fibrosis, energy metabolism and mitochondria, cardio-renal interplay, inflammation, natriuretic peptides and neurohormonal activation. Among them, neurohormonal activation is regarded as a critical regulatory mechanism and now act as a fundamental pharmacological target for the prevention and treatment of adverse remodeling and HF [11]. Emerging therapeutic strategies are backing from neurohormonal systems to the heart muscle, and the potential therapeutic targets such as myocardial hypertrophy, myocardial fibrosis, energy metabolism, etc. are being largely explored in experimental or clinical studies [11–14].

Accumulating evidence showed that post-infarction inflammatory cascade drives the cardiac remodeling and HF following MI [8, 15]. Briefly, sudden necrosis of cardiomyocytes in the infarcted heart results in a release of intracellular contents and initiates an intense inflammation reaction, characterized by the release of amount of pro-inflammatory cytokines. Pro-inflammatory response is further amplified by leukocytes infiltration in the damaged myocardium and contributes to the additional cardiomyocyte death and myocardial injury beyond the original MI zone. An anti-inflammatory reparative phase is then followed (day 4–7), in which fibroblasts and myofibroblasts are recruited and activated resulting in the production of extracellular matrix (ECM) and tissue repair to prevent cardiac rupture. Appropriate inflammatory reaction is necessary for optimal healing of the infarcted heart. However, persistent or chronic inflammation in the infarcted heart induces matrix degradation, finally leading to adverse cardiac remodeling, ventricular dilation, and thereby HF [16]. A lot of experimental and clinical studies have demonstrated that myocardial expression levels of pro-inflammatory cytokines were closely associated with the left ventricular dysfunction, and could act as markers of disease severity and HF prognosis [17, 18]. The absence of TLR4 or NF-κB, two key regulators in the pro-inflammatory cascades, could reduce the extent of LV remodeling and improve HF after MI [19, 20]. Considering the detrimental effects of an excessive and persistent pro-inflammatory response to MI, a potential therapeutic strategy for limiting MI size and preventing adverse LV remodeling is focusing on the attenuation of initial pro-inflammatory response [21, 22].

## Current anti-inflammatory therapies in HF following MI

Up to now, there are no approved anti-inflammatory drugs used to prevent the development of HF following MI. Some cytokines are currently under investigation as potential targets to improve cardiac function and outcome of acute MI (AMI) or chronic HF in experimental models and clinical studies [23]. Generally speaking, anti-cytokine therapies tested in animal models showed beneficial effects on cardiac function and outcome, such as inhibition of IL-1, IL-6, IL-8 and TNF-α, suggesting a promising role of anti-inflammation in the prevention of adverse remodeling after MI [24]. Unfortunately, therapeutic translation from animal studies to human trials has proven disappointing. In smaller clinical studies, anti-cytokine therapy on cardiac function generally obtained favorable results. However, when it comes to larger clinical studies, findings were unsatisfactory in all but only IL-1β [23]. IL-1β blockade with anakinra in one larger study presented positive results in patients with AMI [25]. This role was further demonstrated with canakinumab in larger clinical trials including 10,061 patients with previous MI, in which canakinumab could reduce the incidence of cardiovascular events and the rate of hospitalization due to HF [26, 27].

Several factors hamper the successful translation of experimental research into clinical practice, such as ambivalence of cytokine function, differences in study designs, species, treatment regimens, and chosen endpoints [23, 28]. Although disappointing clinical trials targeting most of cytokines, anti-IL-1β therapy still have brought encouraging signals that anti-inflammatory therapeutic strategies could reduce HF morbidity and mortality [29]. Future therapeutic approaches targeting inflammation-suppression such as multi-targets (combining anti-inflammatory agents with mitochondria and endothelial protective therapies) or novel anti-inflammatory targets (NLRP3 inflammasomes, non-coding RNA, etc.) are under investigation and eagerly awaited [30]. Choosing HF patients with a cardio-inflammatory phenotype would also be helpful to obtain favorable results for the targeted anti-inflammatory strategies [31].

## Traditional Chinese medicine with anti-inflammation activity in MI or HF might act as potential treatments for HF following MI

Traditional Chinese medicines (TCM), with a characteristic of multi-active ingredients, multi-targets and low toxicity, has evolved over the past 5000 years to prevent and manage human disease. The pharmacological activities of TCM are widespread including anti-inflammation. It has been demonstrated that the protective role of TCM in inflammatory diseases is closely related to its anti-inflammatory actions. For example, total glucosides of peony can prevent cerebral ischemia and Alzheimer’s disease by inhibiting inflammatory cytokine production [32]. Tanshinone IIA, with strong anti-inflammatory activity, has been shown to be effective in protecting against MI [33]. The extract of Arisaematis Rhizoma attenuates inflammatory response of paw and joint in the collagen-induced arthritis mice model [34].

Regardless of the aetiology, a lot of experimental evidence has confirmed the participation of inflammation in the development of ischaemic and non-ischaemic HF [2–6]. Various anti-inflammatory therapeutic strategies in different forms of HF have also been assessed in experimental or clinical trials, although most of them failed [2, 7, 8]. TCM which has anti-inflammatory effects might provide some therapeutic hints. Here, we summarized the recent progress about TCM which has anti-inflammatory effects in MI or HF. Among them, single Chinese medicinal herbs (Table 1) and Chinese herbal formulas (Table 2) are discussed separately, in order to provide evidence for their potential application in reducing the onset of HF following MI. These TCM usually suppress multiple pro-inflammatory factors, and also has other regulating activity including cardiac fibrosis, hypertrophy or microcirculation. These features might make them more effective acting as anti-inflammatory strategies in improving HF following MI when compared to single anti-cytokine therapy.

**Table 1:** Single Chinese herbs

Name	In clinic		In experiments		Targets or pathways	Cardiac function	References
	Application	Inflammation	Model	Inflammation			
Astragalus	AMI; congestive HF	IL-6 and TNF-α↓	In LAD-induced MI or HF rats; In ISO-induced CH rats; LPS-induced inflammation injury in H9c2	IL-1β, IL-6, TNF-α, MCP-1 and IL-8↓	TLR4/NF-κB↓ → IκB-α↑; JAK↓; PI3K-AKT↑; miR-127↓	LVIDd and LVIDs↓ LVEF and LVFS↑	[39–41, 46]
Salvia miltiorrhiza	Chronic HF; MI; Stroke	–	In LAD-induced MI rats or mice	IL-1β, IL-6, TNF-α, MCP-1; CD68 and TNF-β↓	NF-κB↓; Trx/JNK↑	LVEF and LVFS↑	[33, 50, 51, 53, 54]
Rhodiola	Chronic MI with HF; MI after PCI	Hs-CRP, IL-6, TNF-α↓	LAD in MI mice; H9C2 cell damage caused by LPS or hypoxic-reoxygenated	IL-6, TNF-α, iNOS, COX-2, PEG2↓	Nox/NF-κB/AP1↓; PI3K/AKT/mTOR↑	LVEDd and LVESd↓	[58, 60]
						LVEF and LVFS↑	[61, 62]
Ge-Gen	CAD; MI;HF	hs-CRP and TNF-α↓	In HF rats induced by TAC; ISO in MI mice	IL-1β, IL-6 and TNF-α↓	PPARα↑ → Nrf1, Fos and Yy1↑; PPAR-γ↑, NF-κB↓	LVEF and LVFS↑	[66, 68–70, 162]
Ginseng	MI; CHF	IL-6 and TNF-α	In MI rats by LAD	MCP-1, TNF-α, IL-1β and IL-6↓	SIRT1↑; IκK/IκB/ NF-κB↓	LVSP↑	[73–75, 77]
						LVEDP↓	
						LV + dp/dt_max_↑	
						LV-dp/dt_min_↓	
Andrographis paniculata	CAD	–	In MI mice by LAD	TNF-α, IL-1β and IL-6↓	NF-κB/MAPK↓, STAT3↑, NF-κB↓	LVEF↑	[81, 82]
						LVESd↓	
Ginkgo	CAD; MI	TLR4/NF-κB, CRP, slCAM-1 and svCAM-1↓	In AMI or HF mice by LAD	TNF-α, IL-1β and IL-6↓	NF-κB↓	LDH, CK and CK-MB↓	[84–88]
Curcumalonga	MI after CABG	–	In MI rats by LAD or ISO;	TNF-α, IL-6, IL-1α, IL-1β↓,	NF-κB↓	CK-MB↓	[92, 94, 95]
Motherwort	Ischemic CAD	–	In ISO-treated CH rats	IL-6, TNF-α, IFN-γ and IFN-1β↓	NF-κB↓	HW/BW and LVW/BW↓	[99–101]
Yanhusso	CAD; HF	–	In HF rats by LAD	TNF-α, IL-1β and IL-6↓	NF-κB↓	LVEDd and LVESd↓	[104–106]
						LVED,LVFS and LVEF↑	
Epimedium	CAD	–	In AMI rats by LAD; In rats with congestive HF	TNF-α, iNOS↓	NF-κB↓	LV + dp/dt_max_↑	[109, 110, 113]
						LV-dp/dt_min_↓	
Schisandra	MI	–	LAD in MI mice; ISO in MI rats	TGF-β1,TNF-α↓, IL-1β, TNF-α, NO, iNOS and PGE2↓	–	LVESd and LVEDd↓	[117, 118]
						LVEF and LVFS↑	
Dragon’s blood	AMI	–	In LAD-induced AMI mice	IL-6↓	IL6-JAK2/STAT3↑	LVEF and LVFS↑	[120]
Lithospermum	–	–	In ISO-induced HF rats; In TAC-induced chronic HF mice	–	TLR4/ NF-κB↓	LVPWd and IVSd↓	[124, 125]
						EF and FS↑

**Table 2:** Chinese herbal formulas

Name	Composition	In clinic		In experiments		Targets or pathway	Cardiac function	Reference
		Application	inflammation	Model	Inflammation			
Gualou Xiebai Decoction	Scallion, Gualouzi	AMI	TGF-β, TNF-α and IL-1↓	LAD in MI rats	∕	NF-κB↓	IVS, LVPW, EF and FS↑	[127–129]
Sini Tang	Aconite, Licorice, Dried Ginger	MI; CAD; CHF	–	LAD in MI rats	IL-6, IL-1β and TNF-α↓	NO↑	EF↑	[130–132]
Qishenyiqi Pill	Salvia Miltiorrhiza, Panax Notoginseng Huang Astragalus, And Dalbergia	HF	–	LAD in MI rats	TNF-α, IL-6, IL-1β↓	LOX↓, NO↑;NF-κB and IL-6-STAT3↓	cTnI↓TLVSP and Max dP/dt↑, LVEDP and Min dP/dt↓	[134–137]
Danhong Injection	Danshen And Honghua	AMI	–	LAD in HF rats	IL-6, IL-1β, TNF-α↓	NF-κB and IκB-α↓	LVEF, LVFS and + dp/dtmax↑ -dp/dtmax↓	[139, 140]
Guan Maitong	Astragalus, Pueraria Salvia Miltiorrhiza, Safflower And Polygonum Multiflorum	CAD	–	LAD in MI rats	IL-1, TNF-α	ICAM-1↓	CK,CK-MB and LDH↓	[142]
Qiliqiangxin	Ginseng Radix Et Rhizoma, Astragali Radix, Aconiti Lateralis Radix Preparata, and et al	CHF	–	LAD in MI rats	TNF-α and IL-6↓; TNF-α/IL-10↓	NF-κB and p-IκBα↓	EF and FS↑, LVEDd↓	[145–148]
Shenfu Injection	Ginseng And Aconite	AMI	–	LAD in HF rats	–	PTX3↓	LVSP, + dp/dt_max_ and -dp/dt_max_↑; LVEDP↓	[149–151]
Shexiang Baoxin Pills	Musk, Ginseng Root, Cow Bezoar, Cinnamon, Cassia Bark, Toad Venom, And Borneol	Angina pectoris	–	LAD in MI rats	IL-6, TNF-α↓	–	LVESP and LV ± *dp*/*dt*_max_↑	[153, 154]
Fufang Danshen Dripping Pill	Danshen, Panax Notoginseng And Borneol	CAD and HF	CRP,IL-6 and TNF-α↓	LAD in AMI rabbits	TNF-α↓	–	–	[160, 161]

### Single Chinese herbs

#### Astragalus

Astragalus is the dried root of *Astragalus membranaceus* or *Astragalus membranaceus Bunge*. It has more than 40 components including saponins, flavonoids, polysaccharides, alkaloids and amino acids, and possess many pharmacological activities such as antitumor, antioxidation and antiinflammation [35]. Astragalus saponins (mainly Astragaloside IV) and Astragalus polysaccharide are the main active components [36, 37]. Astragalus injection is prepared from Radix Astragali and widely used in clinical practice for patients with AMI and congestive HF, acting as an important auxiliary means [38]. Clinical data showed that Astragalus injection could significantly decrease the serum levels of TNF-α, IL-6 and angiotensin II, contributing the improvement of cardiac function in patients with HF [39]. Its anti-inflammation role in MI and HF was also supported by many experimental data. In MI rats induced by the ligation of left anterior descending branch (LAD) [40] and cardiac hypertrophy rats induced by isoproterenol (ISO) [41], Astragaloside IV reduced serum levels of TNF-α and IL-6, and improved cardiac hypertrophy and cardiac function. TLR4/NF-κB signaling pathway is a common pathway for the transcription of inflammatory cytokines and organ failure [42, 43], and mediates the regulation of inflammatory response after MI [44, 45]. Songyi Cheng et al. found that treatment with Astragaloside IV inhibited highly activated inflammatory response by inhibiting TLR4/NF-κB pathway in AMI-induced HF rats [40], indicating it might act as a potential TCM to protect cardiac function after MI by inhibiting inflammation. In vitro, Astragalus polysaccharide reduced myocardial cell damage in a dose-dependent manner and reduced the secretion of inflammatory cytokines, such as IL-6, IL-8, and TNF-α in lipopolysaccharide (LPS)-treated rat cardiomyocytes (H9C2). Its protective effects may be due to the downregulation of miR-127 [46].

#### Salvia miltiorrhiza

Salvia miltiorrhiza is a very popular medicinal plant that has been extensively applied for many years to treat various diseases, either alone or in combination with other Chinese plant-based medicines [47]. It has many biological activities such as antioxidant, neuroprotection, anti-inflammation, anti-atherosclerosis and antitumor [47, 48]. The two main categories of bioactive compounds in Salvia miltiorrhiza are lipophilic constituents (tanshinone I, tanshinone IIa, tanshinone IIb, cryptotanshinone, dihydrotanshinone, etc.) and hydrophilic constituents (danshensu, salvianolic acid A and B, protocatechuic aldehyde, etc.), which synergistically contribute to the cardiovascular protective actions [49]. In a comprehensive review for clinical practice of TCM in chronic HF including 239 Chinese herbs and 1029 papers, salvia miltiorrhiza is listed as the third one with a frequency higher than 300 [50]. It is also clinically used as treatment for ischemic diseases such as MI and stroke [51]. Studies have shown that danshen injection, an extraction of the dried root and rhizome of salvia miltiorrhiza, prevents HF by attenuating post-infarct remodeling [52]. Network pharmacology analysis revealed that salvia miltiorrhiza is used in the cardiovascular disease mainly by inhibiting the inflammatory response [53]. In rats suffering from MI, treatment with tanshinone IIA (Tan IIA) significantly improved systolic and diastolic function. Meanwhile, it reduced the expression level of MCP-1, TNF-α, and macrophage infiltration in rat myocardial tissue. Hence, the cardioprotective effects of Tan IIA may be attributable to its anti-inflammatory properties [33]. The water-soluble derivative of Tan IIA-Tan IIA sodium sulfonate also showed similar protective effect in mice MI model [54]. In addition, the active ingredient salvianolic acid A showed protective role in the cardiac function of MI mice. It could reduce infarct area, improve left ventricular ejection fraction (LVEF), and decrease myocardial levels of inflammatory cytokines IL-1β, IL-6, and TNF-α. The activation of Trx/JNK might mediate this process [55].

#### Rhodiola

Rhodiola (Hong Jing Tian; Crassulaceae) is a genus of medicinal plants that originated in Asia and Europe. The plants and their two major constituents, salidroside and tyrosol, exhibit many bioactivities including antifatigue, antidepressant, antioxidant, anti-inflammation, anticancer, and so on. They play an important role in the prevention of cardiovascular, neuronal, liver, and skin disorders [56–58]. Sofren injection, made from *Rhodiola rosea L.* extract, is commonly used in clinical practice for the treatment of cardiovascular diseases (CVD). The analysis of pharmacological effects in clinical studies has focused on inhibiting excessive inflammatory responses, thereby improving the microenvironment and alleviating the pathological conditions of CVD patients [59]. The clinical efficacy of Rhodiola and its influence on left ventricular remodeling and serum inflammatory mediator level had been evaluated in 46 patients with chronic MI complicated with HF [60]. Results showed that it can effectively inhibit patients’ left ventricular remodeling and alleviate inflammation. Moreover, its injection in AMI patients after percutaneous coronary intervention (PCI) reduced the serum levels of MPO, hs-CRP, IL-6 and TNF-α, and the improved left ventricular function [61]. Experimental data also supported the protective role of Rhodiola in anti-inflammation and cardiac function. In LAD-treated MI mice, treatment with salidroside for 3 weeks can improve LVEF and left ventricular fractional shortening (LVFS), attenuate myocardial inflammation and alleviate the pathological process of myocardial remodeling [62]. In hypoxic-reoxygenated H9C2 cells, pretreatment with salidroside for 1 h also immediately activated the NOx/NF-κB/AP1 pathway to reverse cardiomyocyte damage [63].

#### Ge-gen

Ge-gen is the dried root of Pueraria lobata. It is mainly composed of isoflavones and triterpenes. The triterpene puerarin (gegensu) is the main bioactive component [64, 65]. In a systemic analysis of the clinical efficacy and safety of puerarin as the adjuvant therapy for AMI, puerarin injection could significantly reduce the infarct size, increase the LVEF, and reduce mortality rate [66]. Also, the effect of puerarin injection in HF patients was satisfactory. A meta-analysis based on the randomized controlled trials including 16 RCTs enrolling 1291 HF patients showed that gegensu injection were superior in clinical comprehensive effects including improving LVEF [67] and decreasing serum hs-CRP and TNF-α levels [68]. In HF rats induced by transverse aortic constriction (TAC), puerarin administration reduced the expression of TNF-α, IL-1β and IL-6 in myocardial tissues, and increased expression of PPARα and its related downstream targets Nrf1, Fos, and Yy1. This led to a significant improvement in cardiac function index of LVEF and LVFS [69]. Puerarin derivatives have shown a similar pharmacological effect. In ISO-induced MI mice, Puerarin-V, a novel advantageous crystal form of puerarin, reduced inflammatory cell infiltration, inhibited NF-κB activation, and ultimately resulted in the down-regulation of IL-1β, IL6 and TNF-α [70]. In LPS-treated cardiomyocytes, puerarin could decrease the levels of TNF-α and IL-1β, possibly via the puerarin-induced inhibition of NF-κB signaling [71].

#### Ginseng

Ginseng is the most valuable of medicinal plants, especially in Korea, China, and Japan. Korean ginseng (P. ginseng), Chinese ginseng (Panax notoginseng), and American ginseng (Panax quinquefolius) are the most common ginseng species. Ginseng is the dry root and rhizome of Panax ginseng, and saponins of Panax japonicus are the main active component [72]. It has many bioactivities such as antioxidant, anti-inflammation, antifatigue, antidiabetes, etc. [73]. Clinical data shows that ginseng has a good effect on congestive HF and AMI. In AMI patients, administration of red ginseng extract for 8 months significantly improved coronary flow reserve, reduced serum IL-6 and TNF-α levels, and elevated the absolute numbers of circulating CD34( +), CXCR4( +) and CD117( +) cells [74]. Ginseng decoction or injection also showed improvement of LVEF, left ventricular end-diastolic diameter (LVEDd) and plasma BNP in patients with congestive HF [75, 76]. In LAD ligation induced MI rats, saponins extract from Panax japonicus could significantly improve cardiac function, decrease the serum MCP-1 and TNF-α levels, and suppress the protein expressions of NF-κBp65 subunit, extracellular signal-regulated kinase 1/2 (ERK1/2) and p38 MAPK [77]. Sirt1 plays a key role in regulating the inflammatory response by inhibiting NF-κB signaling and promoting the regression of inflammation [78, 79]. The protective effects of Panax japonicus may be activated by SIRT1 to inhibit NF-κB signaling and inflammation [77].

#### Andrographis paniculata

*Andrographis paniculata* is a medicinal plant traditionally used for the treatment of cold, fever, laryngitis and several infectious diseases. The plant is claimed to possess immunological, antibacterial, anti-inflammatory, antithrombotic and hepatoprotective properties [80]. Its extract contains diterpene, diterpene glycoside, lactone, flavonoids, and flavonoid glycosides. The flavonoid glycoside andrographolide is the major active component [81]. *Andrographis paniculata* has been clinically used to treat patients with coronary artery diseases (CAD) acting as an inhibitor of platelet aggregation. In LAD ligation-induced MI mice, andrographolide showed positive protection against adverse cardiac remodeling after MI and alleviate cardiac dysfunction [82]. Meanwhile, it could reduce the number of inflammatory cells infiltration, protein levels of p-IκBα and p-P65, and the mRNA expression of IL-1, IL-6, TNF-α, and MCP-1 in myocardial tissue [82]. The inhibitory role in inflammation is possibly mediated by the suppression of NF-κB/MAPK signaling pathway[83].

#### Ginkgo biloba

Ginkgo biloba is one of the most ancient medicinal tree species with useful applications in health, food, and supplements. The dry and mature seed of Ginkgo biloba have been clinically used as an antioxidant to inhibit platelet activation, improve vasomotor function, and strengthen the cardiovascular system [84]. The effective components are mainly flavonoids, terpene lactones, and organic acids. Ginkgo Biloba Pills has been reported to inhibit the activation of TLR4/NF-κB inflammatory signaling pathway in peripheral blood of patients with CAD [85]. In patients with AMI, ginkgo biloba extract could down-regulate the activity of macrophage scavenger receptors, and inhibit the serum inflammatory factor levels of CRP, sICAM-1 and sVCAM-1 [86]. Clinical data also showed its protective role in cardiac function of patients with chronic HF [87]. In AMI mice, Ginkgo biloba treatment inhibited infarct size and improved cardiac function, which is mediated by the suppression of inflammation and apoptosis regulating p38 MAPK, NF-κB and Bcl2 signaling pathways [88]. Zhang et al. also found that administration of Ginkgo biloba extract for 4 weeks inhibited TNF-α and IL-1β protein expression, and improved cardiac functions in HF mice [89].

#### Curcuma longa

Curcuma longa is a member of the ginger family (Zingiberaceae), and has rhizomes below the ground. Curcuma longa has been used in a large variety of illnesses, including inflammation, infectious diseases, and gastric, hepatic, and blood disorders. Curcumin is the most abundant component [90]. In 121 patients who underwent coronary artery bypass graft surgery (CABG), curcuminoids treatment can significantly reduce the incidence of MI after CABG, and decrease the level of plasma CRP and malondialdehyde [94]. In LAD-induced rat MI model, curcumin could protect against myocardial ischemia/reperfusion injury through activation of RISK/GSK-3β and inhibition of p38 MAPK and JNK [95]. In ISO-induced rat MI model, curcumin and curcumin nanoparticles both protected cardiac function following MI by reducing pro-inflammatory cytokines levels of TNF-α, IL-6, IL-1α, and IL-1β. Specially, curcumin nanoparticles significantly reduced the expression of MCP-1, prevented myocardial necrosis, and reduced interstitial edema and neutrophil infiltration [96]. However, an overdose of curcumin may cause cardiotoxicity in myocardium possibly due to the inhibition of ion channels [97], which should be carefully considered in the future.

#### Motherwort

Motherwort, also named *Leonurus japonicas*, has excellent therapeutic effects in obstetrical and gynecological diseases. It contains more than 280 compounds, including alkaloids, diterpenes, and flavonoids. Stachydrine is the most abundant alkaloid and active components [98]. Nowadays, motherwort is used for its beneficial effects on the cardiovascular system, and has antibacterial, antioxidant, anti-inflammatory, analgesic, and angiogenic effects [99]. Clinical data have shown that motherwort could improve the cardiac function, microcirculation and hemorheology in patients with ischemic CAD [100]. Animal experiments supported that motherwort protects against CH and fibrosis by suppressing inflammation and oxidative stress [101]. Its active component, stachydrine, could lower serum levels of pro-inflammatory markers of IL-6, TNF-α, IFN-γ and IFN-1β, and down-regulate the expression of p-NF-κB p65 and p-IκBα, leading to the improvement of cardiac hypertrophy in ISO-treated rats [101]. Leonurine is reported to protect cardiac function following AMI through activating the PI3K/AKT/GSK3β signaling pathway [102], which is also the anti-inflammatory pathway [103]. These studies suggested that motherwort might act as a potential TCM to treat HF after MI by inhibiting inflammation.

#### Yanhusuo

Yanhusuo is the dried tuber of *Corydalis yanhusuo*, and has anti-inflammatory, analgesic, and anticancer effects. l-tetrahydropalmatine is the main active component [104]. Yanhusuo has been used to treat CAD in China for a long time, and is also an important component of TCM in treating HF patients [105]. Network pharmacology analysis shows that the key targets of yanhusuo intervention in HF are mainly related to biological processes such as inflammation [105]. The extract from *corydalis yanhusuo* showed beneficial effects in rats with HF following MI, manifested by a significant improvement in infarct size and cardiac function [106]. Its active component of l-tetrahydropalmatine also protected against myocardial ischaemia–reperfusion injury in rats, which could activate the PI3K/Akt/eNOS/NO pathway and increase the expression of HIF-1α and VEGF, resulting in the decrease of inflammatory factors of TNF-α and MPO, therefore contributing to its cardioprotective effect [107].

#### Epimedium

Epimedium is derived from the aerial part of the Epimedium species (Berberidaceae), and has been used in China for over 2000 years to enhance Yang Qi, improve cardiovascular and cerebrovascular functions, and regulate immune function. It has anti-osteoporosis, anti-oxidation, anti-tumor, and anti-aging effects [108]. Compounds such as flavonoid glycoside, phenylpropane, alkaloid, polysaccharides, lignin, and sesquiterpenes have been isolated and identified in *Epimedium*. The most important active compounds are the flavonoid glycosides, including icariin, icariide-ii, epimedin A, and epimedin B [109]. Clinical data showed that Epimedium treatment could significantly improve the symptom of angina, chest congestion, palpitation, breath shortness, as well as the ECG in 120 patients with CAD [110]. In experimental AMI (LAD model) rats, Epimedium flavonoids injection could decrease the area of myocardial infarction, the activity of serum CPK, LDH, and the content of MDA [111]. Icariin, a major component of Epimedium species, could improve rat cardiac ischemia/reperfusion injury by activating the PI3K/Akt/eNOS-dependent signal pathways [112], and reverse ISO-induced rat cardiac injury via inhibiting NF-κB signaling and serum TNF-α level [113]. In rats with congestive HF, ethanol extract from *Epimedium brevicornum* also showed a protective role in cardiac remodeling. It could inhibit cardiomyocyte hypertrophy, cardiomyocyte degeneration and inflammatory infiltration, and decrease the serum levels of TNF-α, norepinephrine, angiotensin II and brain natriuretic peptide [114].

#### Schisandra

Schisandra is the dry ripe fruit of *Schisandra chinensis* (Turcz.) Baill, and has been used in TCM for thousands of years. The pharmacological activities of Schisandra, such as central nervous system stimulation, hepatoprotective effects and anticancer potential, have been confirmed in hundreds of studies [115, 116]. Its active components include schisandrin A/B and schisandra polysaccharides, and the latter is also known as immune bioactive polysaccharides. Schisandra is also a commonly used TCM that has been clinically proven to alleviate the damage of myocytes after the onset of AMI [117]. In ISO-induced rat MI model, Schisandra chinensis bee pollen extract showed cardioprotective effect via antioxidative and anti-apoptosis pathway [118]. In LAD-induced MI mice, schisandrin B could increase survival rate, improve cardiac function and decrease infarct size. Further studies found schisandrin B could down-regulate the expression of hypoxia-induced inflammatory cytokines, such as TGF-β1, TNF-α and NF-κB, which might contribute to its therapeutic effect in ischemic injury [119].

#### Dragon’s blood

One of the sources of dragon's blood is the Chinese dragon's blood (Chinese name: *Longxuejie*), which is derived from the red resins of *Dracaena cochinchinensis* (Lour.) S.C.Chen. It has a variety of therapeutic anti-inflammatory, analgesic, antibacterial, antitumor, and hypoglycemic effects [120]. Loureirin B is the important active component [121]. It has been used in TCM to treat AMI and ischemic heart disease for centuries [122, 123]. Evidence indicated that dragon blood may exert cardio-protective effect by inhibiting inflammatory response during MI, which is supported by experimental data. In LAD-induced AMI mice, the extract of dragon blood significantly improved heart function, and inhibit inflammation via regulating key pathway of IL-6-JAK2/STAT3 in cardiac tissue [122].

#### Lithospermum

Lithospermum is a genus of plants belonging to the family Boraginaceae, herbs or small shrubs*.* It has antibacterial, antifungal, anti-inflammatory, and wound-healing properties [124]. Shikonin is the main bioactive component and isolated from the roots of Lithospermum [125]. In ISO-induced HF mice, Shikonin could reduce myocardial injury and improved cardiac function manifested by increased LVFS. It also inhibited cardiac pro-inflammatory pathways by downregulating the TLR4/NF-κB signaling pathway [126]. In TAC-induced chronic HF mice, its protective role might be partly due to miR-124-medicated attenuation of sympathetic remodeling [127]. At present, there is lacking of clinical evidence of Lithospermum in patients with MI or HF, which is worthy of further evaluation.

### Chinese herbal formulas

#### Gualou Xiebai Decoction

Gualou Xiebai Decoction (GXD) is the alcoholic decoction of gualou and scallion white, among which apigenin and 25S-macrostemonoside P respectively were regard as the major bioactive compounds [128]. It is one of the classical formulas originally recorded in “Jin Kui Yao Lue”, and has been used in CVD for nearly 2000 years [129]. GXD is the commonly used decoction (575 cases, 52.56%) according to an investigation in 1094 patients with AMI from 26 Chinese hospitals [130]. It is also found to inhibit the serum levels of inflammatory factors including TGF-β, TNF-α and IL-1 in 68 patients with AMI [131]. In LAD ligation rats, GXD improved cardiac function, reduced infarct size and pro-inflammatory factor levels (such as TNF-α, IL-1β and NF-κB p65) in infarcted cardiac tissue, indicating that its protective role is mediated by inhibiting the NF-κB associated inflammation [132].

#### Sini Tang

Sini Tang (SNT) is a traditional Chinese herbal formula consisting of four different herbs: the root of Aconitum carmichaelii, the bark of Cinnamomum cassia, the rhizome of Zingiber officinale, and the root of *Glycyrrhiza uralensis*, of which aconitine is the main active components [133]. It has been widely used to improve blood circulation, remove blood stasis, and treat chronic HF, MI or CAD in Chinese clinics [134, 135]. Its anti-inflammatory effect was further demonstrated in MI rats. SNT treatment could significantly decrease the levels of hs-CRP, TNF-α, IL-6, and IL-1β in plasma and myocardial tissue of MI rats, which might contribute to the improvement in cardiac remodeling and heart features [136].

#### Qishenyiqi Pill

Qishenyiqi Pill is a TCM consisting of four different medicinal plants: Salvia miltiorrhiza, Panax notoginseng, Astragalus, and Dalbergia [137], and Astragalus is the master medicine. A lot of clinical data showed that it could decrease all-cause mortality, emergency treatment/hospital admission rate, and improve ventricular remodeling and function in patients with HF. The adverse events were small and uncommon [138, 139]. Experimental data showed that anti-inflammation is involved in its protective role in HF. In LAD ligation rats, Qishenyiqi Pill enhanced left ventricular systolic and diastolic function, and reduced serum levels of IL-6 and TNF-α. It also dose-dependently reduced the expression of phosphorylated-NF-κB and NF-κB, indicating that Qishenyiqi Pill might protect against left ventricular remodeling through inhibition of the NF-κB signaling pathway [140]. It may also act as an anti-inflammatory agent by inhibiting the arachidonic acid LOX pathway and increasing the production of NO [141].

#### Danhong injection

Danhong injection (DHI) is composed of danshen and honghua. Danshen is the monarch medicine and honghua is the minister medicine. DHI is mainly used in the clinical treatment of CVD such as acute coronary syndrome, angina pectoris and cerebrovascular diseases such as stroke in China [142]. The clinical effect of DHI in AMI is also exiting. According to a meta-analysis of randomized controlled trials including 13 RCTs enrolling 979 patients, DHI can protect cardiac function and significantly reduce the risk of HF [143]. Studies have shown that this protective effect is mediated by its anti-inflammation effect. It could decrease the expression of pro-inflammatory cytokines IL-6, IL-1β and TNF-α, and finally improve cardiac function of LAD-induced HF rats. In addition, it inhibited the NF-κB pathway IκB-α in vitro [144].

#### Guan Maitong

Guan Maitong is a traditional Chinese herbal medicine primarily composed of astragalus, pueraria, Salvia miltiorrhiza, safflower, and *Polygonum multiflorum,* of which astragalus and pueraria are both the emperor medicines*.* It has been clinically applied to the treatment of coronary artery disease such as angina pectoris and myocardial ischemia in China [145]. In LAD-induced MI rats, Guan Maitong reduced infarct size, and the levels of CK, CK-MB, and LDH. At the same time, it reduced mRNA and protein expression of IL-1, TNF-α and ICAM-1 in myocardial tissue. It is hypothesized that Guan Maitong had a protective potential against MI injury by inhibiting cardiomyocytes inflammation [146].

#### Qiliqiangxin

Qiliqiangxin consists of Astragalus, ginseng, aconite, Salvia miltiorrhiza, cassava seed, *Alisma orientalis*, *Phyllostachys pubescens*, Guizhi, safflower, Xiangjiapi, and tangerine peel. Astragalus and aconite are both the emperor medicines. It has been used clinically to treat chronic HF [147]. Li et al. conducted a multi-center, randomized, double-blind, parallel group, placebo-controlled experiment. A total of 512 chronic HF patients were enrolled and randomly assigned to receive placebo or Qiliqiangxin*.* The results showed that compared with the placebo group, Qiliqiangxin can significantly increase LVEF, 6-min walk distance and improve quality of life [148]. In LAD ligation rats, Qiliqiangxin significantly reduced left ventricular dilation and improved left ventricular dysfunction. It also reduced the ratio of TNF-α/IL-10 [149] and inhibited NF-κB signaling pathway [150, 151]. Hence, it may improve cardiac function through one of these anti-inflammatory mechanisms.

#### Shenfu injection

Shenfu injection (SFI) consists of ginseng and aconite, and the latter is the master medicine. It has been proven to be safe and effective as a treatment for cardiogenic shock in clinical practice, due to its role in lowering the serum IL-6 level and regulating the equilibrium of pro-inflammatory factors and anti-inflammatory cytokines [152]. SFI could also inhibit inflammation and shorten the disease course in patients with AMI [153], and improve cardiac dysfunction and clinical symptoms during acute aggravation in patients with chronic HF [154]. In HF rats induced by LAD ligation, SPI could decrease inflammatory reactions by targeting haptoglobin and pentraxin 3 [155], contributing to the improvement in hemodynamic function.

#### Shexiang Baoxin Pills

Shexiang Baoxin Pills (SBP) has been widely used in the prevention and treatment of CVDs [156]. It consists of 7 herbal medicines, namely musk, ginseng root, cow bezoar, cinnamon, cassia bark, toad venom, and borneol. Musk is the master one in the patent medicines. Clinically, it can relieve angina pectoris and reduce the prevalence of cardiovascular death and chronic HF [157]. In LAD rats, SBP treatment could improve hemodynamic parameters, inhibit cardiac remodeling, and reduce IL-6 and TNF-α expression [158].

#### Fufang Danshen Dripping Pill

Fufang Danshen Dripping Pill (FDDP) is a famous TCM containing Salvia miltiorrhiza, Radix notoginseng, and Borneol, among which Salvia miltiorrhiza is the master medicine. It is widely used to prevent and treat CAD in China. In a systematic analysis including seven RCTs enrolling 1215 patients, FDDP showed potential benefits for AMI patients, such as the reductions of cardiac death and heart failure [159]. Clinical data also show that FDDP, combined with metoprolol, could improve heart function and reduce serum BNP, CRP and inflammatory factor levels in 120 patients with HF [160]. In LAD-ligated AMI rabbits, FDDP could significantly increase LVEF and LVFS, and improve cardiac function. The possible mechanisms might be related to attenuating local inflammation of myocardium, and inhibiting elevated TNF-α level [161].

## Conclusion

**Fig. 1:**
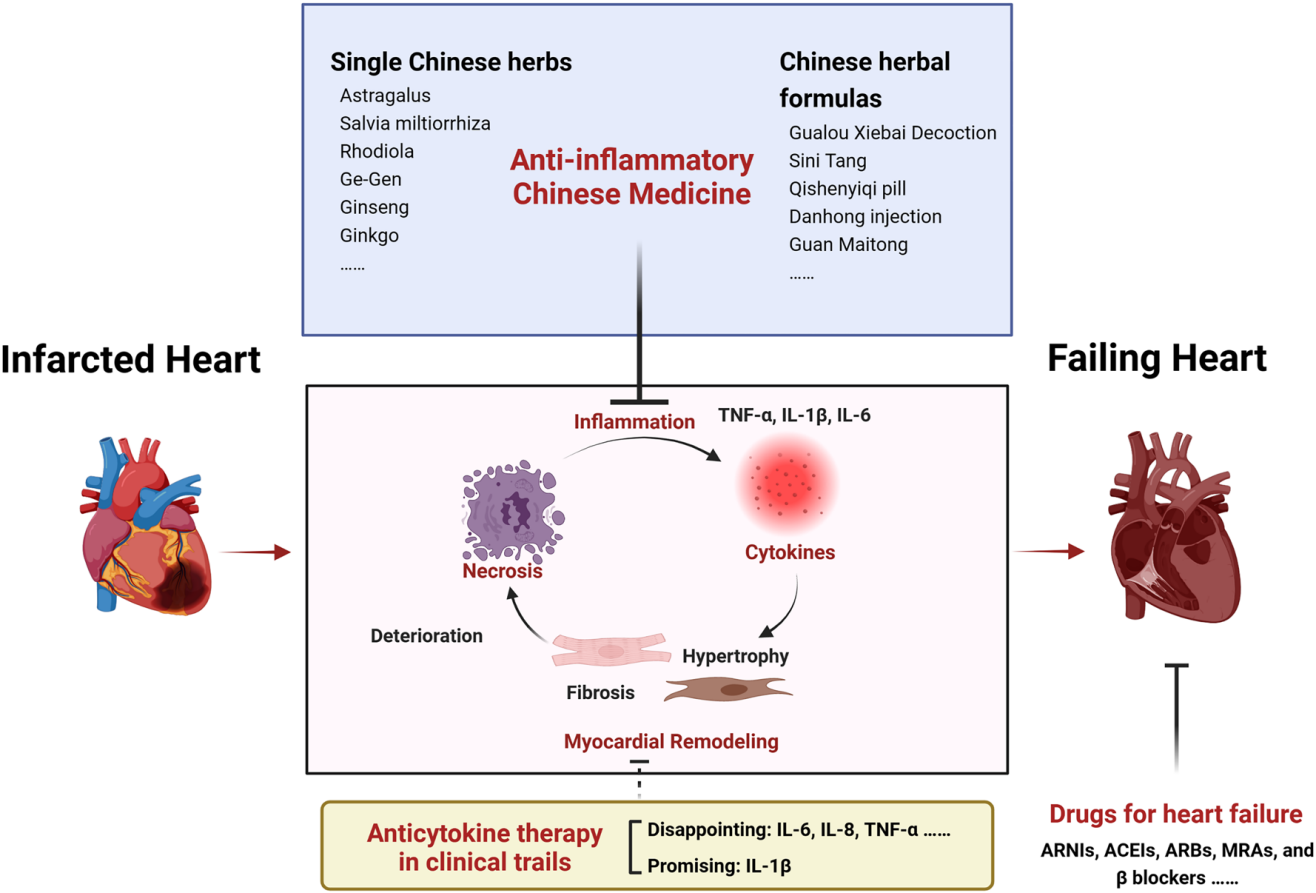
A schematic diagram of anti-inflammatory therapies and existing approved drugs for the treatment of heart failure

HF is a frequent complication of MI. How to delay the onset of HF following MI is an emergency medical problem. Some TCM, with a characteristic of cardiac protection and inflammation inhibition, might play an important role in the process by inhibiting the post-infarction inflammation. Based on clinical application and experimental evidence, we here proposed some TCM, such as Astragalus, Sini Tang, or Qishenyiqi pill, act as the drug candidates (Fig. 1). However, there are still some issues to be resolved: (1) Although TCM is claimed safe and low toxicity, there are still lack of formal data for their adverse effect, which should be explored in the future. (2) The exact anti-inflammatory mechanism remains unclear. Most of studies focus on the phenotype of inflammatory inhibition (eg. inhibition of TNF-α, IL-6, IL-1β or NF-κB). Deep mechanism exploration in the causal relationship should be further clarified. (3) The constituent of TCM is complex. The main active substances and complex interaction between them should also be evaluated. (4) Most of studies focused on the clinical or experimental data from HF or MI. Further studies should be focus on the prevention HF after MI, in order to provide direct evidence for their application in clinic. Moreover, the incidence of HF with preserved ejection fraction (HFpEF) has been increasing in recent years, accounting for almost half of all patients with HF. Due to limited knowledge about the pathophysiological mechanism of HFpEF, effective treatments for HFpEF are still lacking [163]. Anti-inflammatory TCM mentioned here, mainly with the potential to treat HF with reduced ejection fraction (HFrEF), might be worthy of further evaluation in clinical or experimental HFpEF.

## Data Availability

Not applicable.

## Abbreviations

HF: Heart failure
MI: Myocardial infarction
TCM: Traditional Chinese medicine
FAST-MI: French registry of Acute ST elevation or non-ST-elevation Myocardial Infarction
ARNIs: Angiotensin receptor neprilysin inhibitor
ACEIs: Angiotensin-converting enzyme inhibitors
ARBs: Angiotensin receptor blockers
MRAs: Mineralocorticoid receptor antagonists
ECM: Extracellular matrix
LV: Left ventricular
AMI: Acute myocardial infarction
LAD: Left anterior descending branch
ISO: Isoproterenol
LPS: Lipopolysaccharide
LVEF: Left ventricular ejection fraction
PCI: Percutaneous coronary intervention
LVFS: Left ventricular fractional shortening
TAC: Transverse aortic constriction
LVEDd: Left ventricular end-diastolic diameter
CAD: Coronary artery diseases
CABG: Coronary artery bypass graft surgery
HFpEF: Heart failure with preserved ejection fraction
HFrEF: Heart failure with reduced ejection fraction
LVSP: Left ventricular systolic pressure
LVEDP: Left ventricular end diastolic pressure
HW/BW: Heart weight/body weight
LV + dp/dtmax: Maximum left ventricular systolic pressure rate
LV-dp/dtmin: Minimum left ventricular systolic pressure rate
